# Varying Malaria Rapid Diagnostic Test Accuracy by Regional Transmission Level and Demographics in Tanzania

**DOI:** 10.4269/ajtmh.25-0460

**Published:** 2025-12-04

**Authors:** Danielle Wiener, Misago D. Seth, Celine I. Mandara, Rashid A. Madebe, Zachary R. Popkin-Hall, David Giesbrecht, Catherine Bakari, Beatus Lyimo, Dativa Pereus, Filbert Francis, Daniel Mbwambo, Sijenunu Aaron, Abdalah Lusasi, Samwel Lazaro, Timothy P. Sheahan, Jonathan B. Parr, Jeffrey A Bailey, Deus S. Ishengoma, Jonathan J. Juliano

**Affiliations:** ^1^ Department of Epidemiology, Gillings School of Global Public Health, University of North Carolina, Chapel Hill, North Carolina;; ^2^ National Institute for Medical Research, Dar es Salaam, Tanzania;; ^3^ Institute for Global Health and Infectious Diseases, University of North Carolina, Chapel Hill, North Carolina;; ^4^ Brown University, Providence, Rhode Island;; ^5^ Nelson Mandela African Institute of Science and Technology, Arusha, Tanzania;; ^6^ Muhimbili University of Health and Allied Sciences, Dar es Salaam, Tanzania;; ^7^ National Malaria Control Programme, Dodoma, Tanzania;; ^8^ Curriculum in Genetics and Molecular Biology, School of Medicine, University of North Carolina, Chapel Hill, North Carolina;; ^9^ Division of Infectious Diseases, School of Medicine, University of North Carolina, Chapel Hill, North Carolina

## Abstract

Malaria remains a significant global health burden, with ∼263 million cases across 83 countries. It is essential to quickly and accurately detect cases to control the spread of the disease. Given the widespread use of malaria rapid diagnostic tests (mRDTs) for case management and surveillance, it is crucial to understand test reliability. Clarifying how mRDT results differ from real-time polymerase chain reaction (qPCR) test results, as well as the nature of additional variance by test manufacturer, will be useful for reducing measurement bias. After comparing three national standard mRDTs and a research mRDT with qPCR results from a 2021 cross-sectional study in Tanzania, differences were found in terms of age, sex, and regional malaria transmission rate. The research test underperformed overall, with poor sensitivity across transmission strata. After comparing the research mRDT to standard mRDTs, the odds ratios indicated that transmission intensity may affect mRDT agreement and diagnostic performance. These results offer pertinent information on test accuracy and decrease outcome misclassification for malaria prevalence.

## INTRODUCTION

Malaria remains a major public health challenge, affecting ∼263 million people in 2023.^1^ Despite long-standing control efforts, transmission persists. A key strategy in malaria control is test-and-treat, which depends on accurate diagnosis and effective treatment. In Africa, malaria rapid diagnostic tests (mRDTs) are the most widely used diagnostic tool because of their speed, affordability, and ease of use.^1^ Microscopy is less common because of technical and cost barriers, whereas nucleic acid tests like real-time polymerase chain reaction (qPCR) tests offer the highest sensitivity but are impractical for routine use.^2^ However, histidine-rich protein (HRP) 2-based mRDTs face limitations, including inadequate sensitivity that leads to false negatives, false positives due to lingering HRP2 antigen after treatment, and reduced sensitivity to non-falciparum infections.^2^^,^^3^ Additionally, *Pfhrp2* and *Pfhrp3* gene deletions in *Plasmodium falciparum* (*P. falciparum*) have raised concerns about HRP2/3-based mRDT accuracy, as these deletions can result in undetected infections.^4^

Understanding how mRDTs perform across different settings is essential for their effective implementation. Previous studies have revealed that in Southwest Nigeria and the low-transmission areas of Tanzania, the specificity and positive predictive value (PPV) of mRDTs decline among children under 5 years of age, often resulting in overdiagnosis.^2^^,^^5^ Commonly used HRP2/pan-*Plasmodium* lactate dehydrogenase (pLDH) mRDTs have been widely adopted in malaria-endemic countries and generally demonstrate similar diagnostic accuracy.^6^ However, when compared with more sensitive reference methods such as qPCR, these tests tend to exhibit reduced specificity and variable performance, sometimes falling short of WHO standards.^3^^,^^7^^,^^8^

A newer diagnostic tool, the Rapigen Biocredit™ Malaria Ag Pf (Rapigen Inc., Suwon-si, South Korea), utilizes a *Plasmodium falciparum* lactate dehydrogenase (PfLDH)-based approach and has shown promise in detecting malaria cases, particularly in areas where *Pfhrp2* gene deletions are prevalent. However, research on these tests thus far has lacked clarity due to conflicting published results. Some studies have indicated that PfLDH-based mRDTs, such as the Rapigen test, may offer improved sensitivity and specificity in regions where HRP2/3 deletions are common while maintaining comparable accuracy in non-deletion cases compared with PfHRP-based mRDTs.^9^^–^^11^ In contrast, other studies have indicated that PfLDH-based mRDTs are associated with poor sensitivity and specificity compared with qPCR testing, reduced sensitivity compared with PfHRP2 tests, and reduced effectiveness in detecting infections with low parasitemia.^12^^,^^13^ These limitations highlight the need for further research to evaluate the diagnostic accuracy of Rapigen and other PfLDH mRDTs relative to standard HRP2-based tests, especially considering variables such as regional transmission intensity and patient demographics.

The present study is a secondary analysis of 3,284 dried blood spot (DBS) samples collected in 2021 through the Molecular Surveillance of Malaria in Mainland Tanzania project. The original study was conducted to investigate *Pfhrp2/3* gene deletions, population genetics of *Plasmodium* parasites, and the profile of antimalarial drug resistance markers across 10 regions of Tanzania, representing the country’s government-defined malaria transmission strata.^14^^–^^16^ Participants who presented with malaria-like symptoms at health facilities were tested using one or more mRDTs, and DBS samples were collected for molecular analysis. DNA was extracted from the DBS samples using Chelex extraction, and *P. falciparum* was detected using a qPCR assay targeting the 18S ribosomal subunit, as previously described.^17^ Details of the completion of the assays have been previously published in detail, with the data from these studies reused for the current analysis.^4^^,^^18^^–^^20^ Individuals who did not receive both the standard and research mRDTs were excluded from this analysis (final sample: *N* = 3,199; Table 1). Standard mRDTs included the SD Bioline Malaria Ag P.f/pan (#05FK60; Standard Diagnostic Inc., Noida, India), CareStart Malaria HRP2/pLDH (#RMOM-02571; AccessBio Inc., Monroe Township, NJ), and First Response Malaria Ag HRP2/pLDH Combo (#PI16FRC10s; Premier Medical Corp., Valsad, India) tests. The research mRDT evaluated was the BIOCREDIT Malaria Ag Pf test (Pf-pLDH; #C14RHG25; Rapigen Inc.).

**Table 1:** Descriptive characteristics of the study sample, Tanzania 2021 (*N* = 3,199)

Variable	Range	Mean (SD)	*n* (%)*	Missing Values, *n* (%)
qPCR results				
Positive for malaria	–	–	1,833 (57.3)	0 (0)
Negative for malaria	–	–	1,366 (42.7)	
mRDT results by test^†^	–	–	–	0 (0)
Care Start (national standard; 380)				
Positive for malaria	–	–	230 (60.5)	–
Negative for malaria	–	–	150 (39.5)	–
First Response (national standard; 1,848)				
Positive for malaria	–	–	900 (48.7)	–
Negative for malaria	–	–	948 (51.3)	–
SD Bioline (national standard; 967)				
Positive for malaria	–	–	580 (60.0)	–
Negative for malaria	–	–	387 (40.0)	–
Rapigen (research mRDT; 2,000)				
Positive for *P. falciparum* malaria	–	–	805 (40.3)	–
Negative for *P. falciparum* malaria	–	–	1,195 (59.8)	–
Sex				
Male	–	–	1,462 (45.7)	0 (0)
Female	–	–	1,737 (54.3)	
Geographical transmission strata				
Very low	–	–	1,328 (41.5)	0 (0)
Low	–	–	614 (19.2)	
Moderate	–	–	551 (17.2)	
High	–	–	706 (22.1)	
Age (years)	0–99	18.65 (19.33)	–	5 (0.002)
Age group (years)				
Child (<5)	–	–	1,190 (37.2)	5 (0.002)
School-aged (5–16)	–	–	618 (19.3)	
Adult (>16)	–	–	1,386 (43.3)	

mRDT = malaria rapid diagnostic test; *P. falciparum* = *Plasmodium falciparum*; qPCR = real-time polymerase chain reaction.

* Percentage of non-missing observations.

^†^ Followed by type (national standard versus research test) and the total number of each mRDT type administered in the sample. Of those in the sample, 3,195 (99.9%) received one of the three options for a national standard mRDT.

Test accuracy was assessed using sensitivity, specificity, PPV, and negative predictive value (NPV). Because the PPV and NPV depend on disease prevalence, they are not generalizable across settings with different malaria rates. Confidence intervals for diagnostic test accuracy estimates were included in Tables 2 and 3 to increase reliability. A supplementary analysis involved the application of a parasitemia threshold to qPCR results, treating samples with ≤50 copies as negative, reflecting the typical detection limit of mRDTs and expert microscopy. Results from this threshold-based analysis are presented in Supplemental Tables 1 and 2, alongside unadjusted results. Logistic regression with SAS 9.4 (SAS Institute, Cary, NC) was used to calculate diagnostic odds ratios (ORs) for agreement between the research and standard mRDTs, stratified by transmission intensity (high, moderate, low, or very low) on the basis of Tanzania’s 2020 malaria stratification data.^14^

**Table 2:** Accuracy of malaria rapid diagnostic tests compared with real-time polymerase chain reaction tests by sex and age group

Crude Analyses	Sensitivity	Specificity	PPV*	NPV*
mRDT Type				
Care Start	0.876 (0.834–0.917)	0.908 (0.859–0.958)	0.948 (0.919–0.977)	0.793 (0.729–0.858)
First Response	0.829 (0.805–0.852)	0.876 (0.855–0.898)	0.877 (0.855–0.898)	0.828 (0.804–0.852)
SD Bioline	0.868 (0.842–0.895)	0.899 (0.867–0.931)	0.941 (0.922–0.961)	0.786 (0.745–0.826)
Rapigen	0.734 (0.708–0.761)	0.975 (0.965–0.985)	0.971 (0.960–0.983)	0.763 (0.739–0.787)
Stratified by biological sex				
Male				
Care Start	0.870 (0.804–0.936)	0.915 (0.844–0.986)	0.946 (0.899–0.992)	0.806 (0.711–0.901)
First Response	0.832 (0.797–0.867)	0.871 (0.838–0.903)	0.874 (0.842–0.906)	0.827 (0.791–0.863)
SD Bioline	0.890 (0.856–0.924)	0.870 (0.815–0.924)	0.937 (0.910–0.964)	0.784 (0.721–0.847)
Rapigen	0.759 (0.721–0.796)	0.967 (0.950–0.984)	0.964 (0.946–0.983)	0.773 (0.737–0.809)
Female				
Care Start	0.879 (0.827–0.932)	0.903 (0.834–0.971)	0.949 (0.913–0.986)	0.783 (0.694–0.872)
First Response	0.826 (0.793–0.859)	0.881 (0.852–0.909)	0.879 (0.850–0.908)	0.829 (0.796–0.861)
SD Bioline	0.846 (0.806–0.886)	0.922 (0.884–0.960)	0.946 (0.919–0.973)	0.787 (0.733–0.840)
Rapigen	0.713 (0.676–0.750)	0.982 (0.971–0.994)	0.978 (0.964–0.992)	0.755 (0.723–0.788)
Stratified by age group				
Children (<5 years)				
Care Start	0.929 (0.875–0.984)	0.850 (0.739–0.961)	0.929 (0.875–0.984)	0.850 (0.739–0.961)
First Response	0.848 (0.814–0.883)	0.834 (0.795–0.874)	0.861 (0.827–0.894)	0.820 (0.780–0.860)
SD Bioline	0.863 (0.816–0.910)	0.871 (0.806–0.937)	0.931 (0.895–0.967)	0.759 (0.681–0.836)
Rapigen	0.697 (0.651–0.742)	0.978 (0.962–0.994)	0.975 (0.956–0.993)	0.728 (0.686–0.770)
School-aged (5–16 years)^†^				
Care Start	0.864 (0.790–0.939)	0.886 (0.780–0.991)	0.946 (0.894–0.997)	0.738 (0.605–0.871)
First Response	0.866 (0.816–0.916)	0.844 (0.771–0.916)	0.912 (0.869–0.954)	0.771 (0.691–0.852)
SD Bioline	0.883 (0.835–0.931)	0.778 (0.667–0.889)	0.926 (0.886–0.966)	0.677 (0.561–0.794)
Rapigen	0.816 (0.767–0.864)	0.952 (0.915–0.990)	0.971 (0.948–0.994)	0.727 (0.659–0.795)
Adult (>16 years)				
Care Start	0.831 (0.751–0.912)	0.964 (0.916–1.000)	0.972 (0.933–1.000)	0.794 (0.698–0.890)
First Response	0.787 (0.744–0.829)	0.914 (0.888–0.940)	0.878 (0.842–0.914)	0.845 (0.813–0.877)
SD Bioline	0.862 (0.819–0.904)	0.951 (0.919–0.982)	0.960 (0.935–0.986)	0.833 (0.782–0.883)
Rapigen	0.721 (0.679–0.763)	0.979 (0.967–0.992)	0.969 (0.950–0.988)	0.799 (0.766–0.831)

mRDT = malaria rapid diagnostic test; NPV = negative predictive value; PPV = positive predictive value. 95% CIs are included in parentheses for each estimate.

* For these analyses, the prevalence of the usage of mRDTs compared with real-time polymerase chain reaction tests in the sample population is inherent to the calculation. For a population with varying levels of mRDT administration, the PPV and NPV will be different.

^†^ Based on the age range used in the 2017 Tanzania National School Children Survey.

**Table 3:** Accuracy of malaria rapid diagnostic tests compared with real-time polymerase chain reaction tests by transmission strata

mRDT Type	Sensitivity	Specificity	PPV*	NPV*
Very low				
Care Start^†^	0.783 (0.614–0.951)	0.986 (0.958–1.000)	0.947 (0.847–1.000)	0.932 (0.875–0.990)
First Response	0.635 (0.578–0.692)	0.916 (0.893–0.938)	0.777 (0.722–0.831)	0.844 (0.816–0.872)
SD Bioline	0.866 (0.818–0.914)	0.931 (0.892–0.969)	0.933 (0.897–0.970)	0.861 (0.811–0.911)
Rapigen	0.655 (0.606–0.703)	0.987 (0.979–0.996)	0.968 (0.946–0.990)	0.831 (0.804–0.858)
Low				
Care Start^†^	0.933 (0.880–0.985)	0.920 (0.814–1.000)	0.976 (0.944–1.000)	0.793 (0.646–0.941)
First Response	0.893 (0.844–0.943)	0.826 (0.749–0.904)	0.893 (0.844–0.943)	0.826 (0.749–0.904)
SD Bioline	0.871 (0.822–0.920)	0.897 (0.830–0.965)	0.951 (0.918–0.984)	0.753 (0.665–0.840)
Rapigen	0.816 (0.769–0.862)	0.968 (0.940–0.996)	0.977 (0.958–0.997)	0.754 (0.694–0.814)
Moderate				
Care Start^†^	0.907 (0.849–0.965)	0.667 (0.449–0.884)	0.936 (0.887–0.986)	0.571 (0.360–0.783)
First Response	0.931 (0.893–0.969)	0.863 (0.768–0.957)	0.959 (0.926–0.989)	0.786 (0.678–0.893)
SD Bioline	0.841 (0.783–0.899)	0.883 (0.802–0.965)	0.948 (0.910–0.985)	0.688 (0.585–0.792)
Rapigen	0.823 (0.772–0.874)	0.913 (0.847–0.980)	0.967 (0.941–0.993)	0.624 (0.529–0.718)
High				
Care Start^†^	0.725 (0.587–0.863)	0.833 (0.661–1.000)	0.906 (0.805–1.000)	0.577 (0.387–0.767)
First Response	0.901 (0.870–0.932)	0.763 (0.697–0.828)	0.894 (0.862–0.926)	0.777 (0.712–0.842)
SD Bioline^†^	0.906 (0.850–0.961)	0.741 (0.575–0.906)	0.932 (0.883–0.981)	0.667 (0.498–0.835)
Rapigen	0.681 (0.618–0.743)	0.950 (0.902–0.998)	0.974 (0.948–0.999)	0.524 (0.443–0.605)

mRDT = malaria rapid diagnostic test; NPV = negative predictive value; PPV = positive predictive value.

* For these analyses, the prevalence of the usage of mRDTs compared with real-time polymerase chain reaction tests in the sample population is inherent to the calculation. For a population with varying levels of mRDT administration, the PPV and NPV will be different.

^†^ The following strata had smaller sample sizes: very low Care Start (*n* = 93), low Care Start (*n* = 114), moderate Care Start (*n* = 115), high Care Start (*n* = 58), and high SD Bioline (*n* = 133).

When stratified by age and sex, test accuracy varied by manufacturer (Table 2). The research mRDT (Rapigen PfLDH) exhibited the lowest sensitivity across all age groups, performing worst in each stratum. Among children under 5 years of age, CareStart had the highest sensitivity (92.9%), whereas Rapigen had the lowest (69.7%). Specificity was highest among adults aged 17 years and older (91.4–97.9%) across all tests and remained consistently high for the research mRDT across age and sex. The PPV was generally high across all strata, with First Response being the only test for which the PPV consistently fell below 0.900. In contrast, the NPV was the weakest metric overall, with 27 of 40 estimates exhibiting an NPV below 0.800. The research mRDT had the lowest NPV in nearly every group. Overall, demographic stratification revealed that although the research mRDT had relatively high specificity, it consistently underperformed in sensitivity.

**Figure 1.**
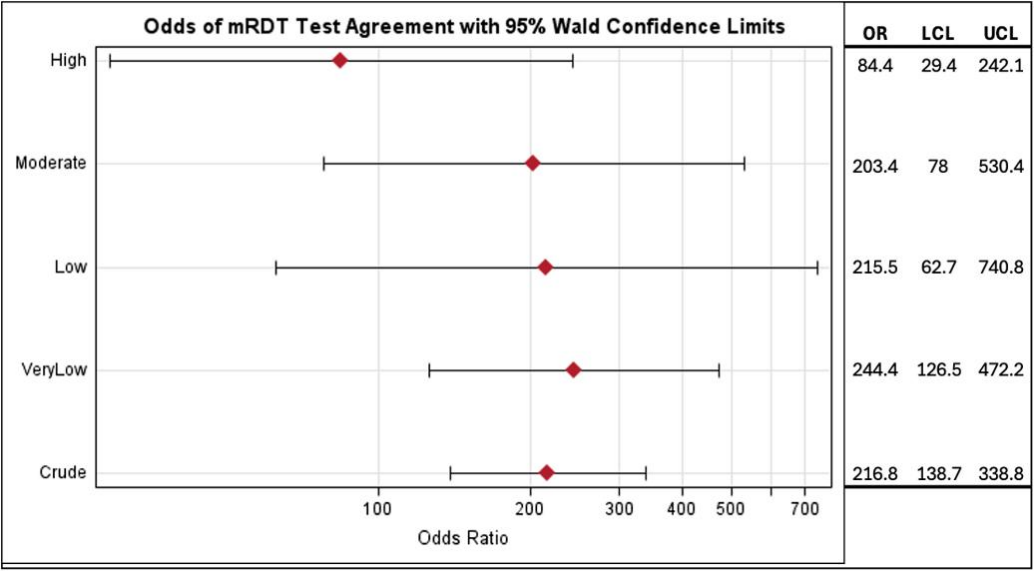
Odds ratio for agreement between Rapigen test and standard malaria rapid diagnostic tests (mRDTs). The likelihood of agreement between the research Rapigen test and standard mRDTs is shown stratified by transmission intensity. LCL = lower confidence interval; OR = odds ratio; UCL = upper confidence interval. All ORs have a *P*-value <0.0001.

Regional malaria transmission levels appeared to influence mRDT accuracy, with variation by test manufacturer. CareStart and First Response exhibited higher sensitivity in low- to moderate-transmission areas, whereas First Response performed poorly in very low-transmission regions (sensitivity = 63.5%; PPV = 77.7%). SD Bioline exhibited reduced specificity in high-transmission areas (74.1%). The PPV remained high across all strata and tests, whereas the NPV was generally low, except in very low-transmission areas. Although diagnostic ORs for agreement between the research and standard mRDTs varied by region, overlapping CIs suggested a limited statistical difference (Figure 1). Overall, the odds of a positive malaria diagnosis using the standard mRDTs among those who were diagnosed positive using the PfLDH-based test were 216.8 (95% CI: 138.7–338.8) times the odds of those who were diagnosed negative using the PfLDH-based test, indicating high sensitivity and agreement. In very low- to moderate-transmission regions, the odds of a positive result on a standard test given a positive research test ranged from 203.4 to 244.4 (95% CI: 62.7–740.8). The high-transmission region exhibited a lower OR estimate (84.4; 95% CI: 29.4–242.1), suggesting that transmission intensity may affect mRDT agreement and diagnostic performance.

Applying a qPCR parasitemia threshold of ≥50 copies (Supplemental Tables 1 and 2) did not meaningfully alter the results, with accuracy estimates changing by no more than 2 percentage points; the only exception was the CareStart mRDT, which had a smaller sample size (*n* = 380). This limited sample may reduce the reliability of its accuracy estimates.

Key limitations of the present study include the small sample size for CareStart and wide CIs from logistic regression, indicating lower precision. A larger sample would likely improve estimate stability, as seen in the overall crude OR (Figure 1) compared with the stratified analyses. Additionally, all samples were collected from symptomatic individuals, which limits generalizability to asymptomatic or non-health-seeking populations. Although a primary use of mRDTs is for diagnosing symptomatic malaria, they can also be used for various research or surveillance purposes. In those cases, further research is needed to elucidate diagnostic reliability among such groups.

Another possible issue is a possible underlying inequivalence in disease prevalence among the mRDT analysis groups. The results of a Kruskal–Wallis test indicated that among those administered a national standard mRDT, there was a significant difference in median parasitemia value (χ^2^ = 12.41; *P* = 0.002; df = 2). However, further examination using the Dwass, Steel, and Critchlow–Fligner multiple comparison procedure indicates that the only between-group significant differences occurred when comparing the SD Bioline and First Response test groups (*P* = 0.0013). There is some variation in which national standard mRDT was used by region, but the percentage of participants in each region who were additionally randomly selected for testing with Rapigen was relatively consistent (Supplemental Table 3). The number of test samples by mRDT type, stratified by transmission strata, is shown in Supplemental Table 4, which reveals similar variation by national standard and consistency of analytical sampling for Rapigen.

The present study highlights notable differences in diagnostic performance between standard mRDTs and the newer Rapigen test. Although Rapigen exhibited high specificity—potentially reducing overtreatment—it consistently underperformed in terms of sensitivity and NPV, especially in high-transmission areas. These findings are concerning, as missed infections can lead to untreated cases and sustained transmission. Accurate diagnosis is essential for malaria control, particularly because *Pfhrp2/3* gene deletions and rising drug resistance challenge current strategies. Whereas sensitivity ensures timely treatment, specificity helps avoid unnecessary drug use and resistance. A reliable diagnostic test must perform well across diverse populations and settings. These findings underscore the need for ongoing evaluation of mRDTs as malaria transmission patterns and parasite genetics evolve. Strengthening diagnostic tools is critical to support test-and-treat programs and advance toward malaria elimination goals.

## Supplemental Materials

10.4269/ajtmh.25-0460 Supplemental Materials
