# Supplementary material for: Varying Malaria Rapid Diagnostic Test Accuracy by Regional Transmission Level and Demographics in Tanzania
Source: Am J Trop Med Hyg. 2025 Dec 4;114(2):287–91. doi: 10.4269/ajtmh.25-0460 (PMC12874920; doi:10.4269/ajtmh.25-0460)
Supplement: Supplemental Materials [file tpmd250460.SD1.pdf]

Supplemental Table 1. Test Accuracy of mRDTs compared to qPCR (qPCR parasitemia cutoff below 50)

mRDT Test Type	Sensitivity	Specificity	Positive Predictive Value (PPV)*	Negative Predictive Value (NPV)*
Crude Analyses				
Care Start	0.874	0.888	0.935	0.793
First Response	0.830	0.876	0.877	0.892
SD Bioline	0.871	0.897	0.940	0.791
Rapigen	0.735	0.973	0.969	0.765
Stratified by Biological Sex				
Male				
Care Start	0.869	0.900	0.935	0.806
First Response	0.832	0.871	0.874	0.827
SD Bioline	0.892	0.865	0.934	0.790
Rapigen	0.760	0.965	0.962	0.775
Female				
Care Start	0.878	0.878	0.945	0.783
First Response	0.828	0.881	0.879	0.830
SD Bioline	0.848	0.922	0.946	0.791
Rapigen	0.714	0.981	0.976	0.757
Stratified by Age Group				
Children (<5)				
Care Start	0.928	0.810	0.906	0.850
First Response	0.848	0.834	0.860	0.820
SD Bioline	0.867	0.873	0.931	0.767
Rapigen	0.697	0.972	0.968	0.730
School Aged (5-16) †				
Care Start	0.864	0.886	0.946	0.738

First Response	0.871	0.845	0.912	0.781
SD Bioline	0.882	0.764	0.920	0.677
Rapigen	0.816	0.952	0.971	0.727
Adult (>16)				
Care Start	0.829	0.946	0.958	0.791
First Response	0.787	0.914	0.878	0.845
SD Bioline	0.865	0.951	0.960	0.837
Rapigen	0.723	0.979	0.969	0.800

*For these analyses, the prevalence of the usage of mRDT tests compared to qPCR in the sample population is inherent to the calculation. For a population with differing levels of mRDT test administration, the PPV and NPV will be different.

† Based on the age range used in the 2017 Tanzania National School Children Survey

Supplemental Table 2. mRDT Accuracy v. qPCR, Stratified by Malaria Transmission Levels in Tanzania 2021 with qPCR Parasitemia Threshold

mRDT Test Type	Sensitivity	Specificity	Positive Predictive Value (PPV)*	Negative Predictive Value (NPV)*
Very Low				
Care Start†	0.783	0.986	0.947	0.932
First Response	0.635	0.916	0.777	0.844
SD Bioline	0.875	0.931	0.933	0.872
Rapigen	0.658	0.987	0.968	0.833
Low				
Care Start†	0.932	0.885	0.965	0.793
First Response	0.893	0.826	0.893	0.826
SD Bioline	0.871	0.897	0.951	0.753
Rapigen	0.815	0.962	0.973	0.754
Moderate				
Care Start†	0.905	0.600	0.915	0.571
First Response	0.931	0.863	0.959	0.786
SD Bioline	0.840	0.869	0.940	0.688
Rapigen	0.822	0.900	0.962	0.624
High				
Care Start†	0.725	0.833	0.906	0.577
First Response†	0.904	0.764	0.894	0.783
SD Bioline	0.906	0.740	0.932	0.667
Rapigen	0.681	0.950	0.974	0.524

*For these analyses, the prevalence of the usage of mRDT tests compared to qPCR in the sample population is inherent to the calculation. For a population with differing levels of mRDT test administration, the PPV and NPV will be different.

† These strata had small sample sizes; Very Low Care Start (n = 93), Low Care Start (n=114), Moderate Care Start (n = 115), High Care Start (n = 58), High SD Bioline (n = 133)

Supplemental Table 3. Number of Test Samples by mRDT type, Stratified by Transmission Strata

mRDT Test Type	Number of Samples Tested	% of Samples within Strata
Very Low		
Care Start	93	4.0
First Response	867	37.3
SD Bioline	367	15.8
Rapigen	999	42.9
Low		
Care Start	114	11.0
First Response	242	23.4
SD Bioline	256	24.8
Rapigen	421	40.8
Moderate		
Care Start	115	13.8
First Response	225	26.9
SD Bioline	211	25.3
Rapigen	284	34.0
High		
Care Start	58	5.8
First Response	514	51.3
SD Bioline	133	13.3
Rapigen	296	29.6

Supplemental Table 4. Number of Test Samples by mRDT type, Stratified by Region

mRDT Test Type	Number of Samples Tested	% of Samples in Region
Dar es Salaam		
Care Start	53	15.2
First Response	21	6.0
SD Bioline	138	39.7
Rapigen	136	39.1
Dodoma		
Care Start	4	0.8
First Response	150	32.0
SD Bioline	118	25.2
Rapigen	197	42.0
Kagera		
Care Start	55	9.5
First Response	356	61.5
SD Bioline	0	0.0
Rapigen	168	29.0
Kilimanjaro		
Care Start	64	4.0
First Response	721	45.7
SD Bioline	112	7.1
Rapigen	681	43.2
Manyara		
Care Start	12	3.2
First Response	121	32.5
SD Bioline	87	23.4

Rapigen	152	40.9
Mara		
Care Start	78	18.7
First Response	57	13.7
SD Bioline	149	35.7
Rapigen	133	31.9
Mtwara		
Care Start	3	0.7
First Response	158	37.5
SD Bioline	133	31.5
Rapigen	128	30.3
Njombe		
Care Start	17	4.5
First Response	25	6.7
SD Bioline	168	44.7
Rapigen	166	44.1
Songwe		
Care Start	57	26.4
First Response	71	32.9
SD Bioline	0	0.0
Rapigen	88	40.7
Tabora		
Care Start	37	8.9
First Response	168	40.2
SD Bioline	62	14.8
Rapigen	151	36.1
